# Combined Effect of TID Radiation and Electrical Stress on NMOSFETs

**DOI:** 10.3390/mi13111860

**Published:** 2022-10-29

**Authors:** Yanrong Cao, Min Wang, Xuefeng Zheng, Enxia Zhang, Ling Lv, Liang Wang, Maodan Ma, Hanghang Lv, Zhiheng Wang, Yongkun Wang, Wenchao Tian, Xiaohua Ma, Yue Hao

**Affiliations:** 1 School of Electronics & Mechanical Engineering, Xidian University, Xi’an 710071, China; 2 State Key Discipline Laboratory of Wide Bandgap Semiconductor Technology, School of Microelectronics, Xidian University, Xi’an 710071, China; 3 Department of Electrical Engineering and Computer Science, Vanderbilt University, Nashville, TN 37235, USA; 4 Beijing Microelectronics Technology Institute, Beijing 100076, China

**Keywords:** TID, electrical stress, combined effect, NMOSFETs

## Abstract

The combined effect of total ionizing dose (TID) and electrical stress is investigated on NMOSFETs. For devices bearing both radiation and electrical stress, the threshold voltage shift is smaller than those only bearing electrical stress, indicating that the combined effect alleviates the degradation of the devices. The H bond is broken during the radiation process, which reduces the participation of H atoms in the later stage of electrical stress, thereby reducing the degradation caused by electrical stress. The positive charges of the oxide layer generated by radiation neutralize part of the tunneling electrons caused by electrical stress, and consume some of the electrons that react with the H bond, resulting in weaker degradation. In addition, the positive charges in shallow trench isolation (STI) generated by radiation create parasitic leakage paths at the interfaces of STI/Si, which increase the leakage current and reduce the positive shift of the threshold voltage. The parasitic effect generated by the positive charges of STI makes the threshold voltage of the narrow-channel device degrade more, and due to the gate edge effect, the threshold voltage of short-channel devices degrades more.

## 1. Introduction

With the development of semiconductor technology, the aerospace applications of integrated circuits have been greatly developed, and the extreme environment of space has seriously affected the operation of devices. Statistics show that the electronic circuits in satellites have more failures than mechanical failures, accounting for 54% of the total [1]. Unfavorable factors such as space radiation seriously affect the normal operation of the devices. Under space conditions, MOS devices will endure multiple radiation effects, e.g., single-event effects (SEEs) and total ionizing dose (TID) effects. In terms of total ionizing dose effects, much research has been conducted internationally, and it is generally believed that changes in parameters, such as the threshold voltage (*V_th_*) of MOS devices after radiation, are mainly caused by the positive charges of the oxide layer and the interface traps at the SiO_2_/Si interface [2,3]. In addition, with the thinning of the gate oxide layer, the impact of shallow trench isolation (STI) becomes more significant [4,5,6]. The device reliability after radiation is also important for applications. Electrical stress on MOS devices causes a shift in threshold voltage, degradation in transconductance, etc. Changes in these device parameters may cause analog and digital circuit failures [7,8,9,10]. High-K gate dielectrics have been widely used in current technologies, which may lead to more severe degradations in NMOSFETs under positive gate bias [11]. Katsunori Onishi et al. believe that the NMOS degradation caused by positive gate stress was mainly due to the charges trapped in the HfO_2_ layer, rather than the interface trap [12,13]. N. Sa et al. proposed an improved version of the R-D model to explain the negative shift mechanism of threshold voltage during stress, which was attributed to the broken Si-O bond and the diffusion of O- in the HfO_2_ layer [14]. Mukhopadhyay et al. believed that the main reason for the degradation of high-K gate devices was the H-bond broken at the interface of HfO_2_/SiO_2_ and H- diffusion in the HfO_2_ layer due to the strong electric field [15]. Although there are many studies on the radiation or electrical stress of MOS devices, few studies have been reported on the combined effect of radiation and electrical stress on MOS devices. These combined effects may have new influences on the reliability of these devices. Therefore, we conducted in-depth research on the influence of the combined effects of radiation and electrical stress on device reliability. In this paper, we first analyze the combined effect of radiation and electrical stress on the characteristics of devices, discuss the effect of device structure size on the combined effect, and then determine the effects of parasitic gate and gate edge on the device.

## 2. Experiments

The experimental NMOSFETs samples were fabricated with a 28 nm process; the ratios of width and length were 1.5 μm/0.55 μm, 1.5 μm/1.1 μm, and 2.5 μm/0.55 μm, with EOT = 6.19 nm and operation voltage = 3.3 V. The selected devices were divided into two groups. The first group was for total ionizing dose radiation + electrical stress tests, and the second group was for electrical stress only. The radiation experiment was conducted at the Northwest Institute of Nuclear Technology. The radiation source was ^60^Co γ-rays and the dose rate was 61 rad (Si)/s. The total dose of 1000 krad(Si) was radiated to the NMOSFETs; the bias during radiation was ON state with gate voltage of *V_g_* = 3.3 V, and V_s_ = V_d_ = V_sub_ = 0 V. Electrical stress experiments were performed after a total dose of 1000 krad(Si). During stress, gate voltage was 4.5 V (V_gstress_ = 4.5 V) and V_s_ = V_d_ = V_sub_ = 0 V. The electrical characteristics of the devices were tested at different time points. The total stress time was 1000 s, and the recovery time was 1000 s with *V_g_* = V_s_ = V_d_ = V_sub_ = 0 V.

## 3. Experimental Results and Discussion

### 3.1. Initial I–V Characteristics of the Device

**Figure 1:**
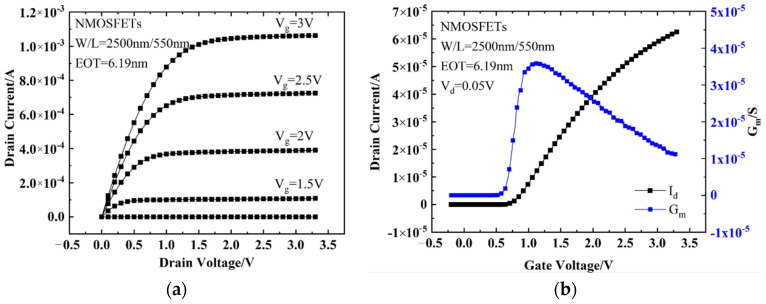
I−V characteristics for a typical NMOSFET in this work: (**a**) output characteristics and (**b**) transfer and transconductance curves.

The initial characteristics of the devices were tested and the results are shown in Figure 1. The threshold voltage obtained from the transfer characteristics is *V_th_* = 0.717 V, and the maximum transconductance is *G_m-max_* = 3.63 × 10^−5^ S. The threshold voltage here is calculated using the constant current method [16], and the expression is shown below, where *W* and *L* are the gate width and gate length of NMOS devices, respectively. All the parameters are normal.

(1) $$V_{th}=V_{g}@0.1\times \frac{W}{L}(\mu A)\;$$

### 3.2. Combined Effect of Radiation and Electrical Stress

#### 3.2.1. Effect of Radiation on I–V Characteristics

**Figure 2:**
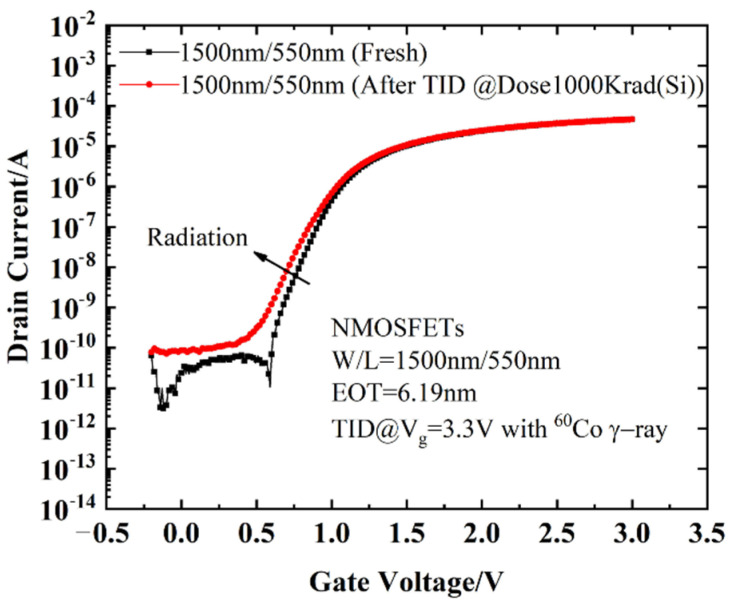
Drain current as a function of gate voltage for devices before and after TID radiation. The *W*/*L* of the devices is 1.5 µm/0.55 µm.

Figure 2 is a comparison of the transfer characteristics for NMOS devices before and after TID. The threshold voltage slightly shifted negatively and the subthreshold slope stretched out slightly after TID due to the positive charge generated in the oxide layer and the interface traps building up during irradiation [2,17]. In addition, device degradation is related to the HfO_2_ layer and shallow trench isolation (STI) [6,18]. The threshold voltage shift is composed of the oxide layer charges and the interface traps; the expression is [18]:

(2) $$\Delta V_{th}=\Delta V_{ot}+\Delta V_{it}=\frac{t_{ox}}{\varepsilon_{0}\varepsilon_{ox}}\cdot q\left(\Delta N_{it}-\Delta N_{ot}\right),$$

where *q* is the electron charge, *ε_ox_* is the dielectric constant of the oxide, *t_ox_* is the thickness of the oxide layer, Δ*N_ot_* is the positive trap charge density of the oxide layer, and Δ*N_it_* is the interface trap charge density.

The calculation of interface traps is expressed by the following formula [19]:

(3) $$\Delta N_{it}{}_{\;}=\frac{1}{\mathrm{lg}\left(kT\right)}\cdot \frac{C_{ox}}{q}\cdot \Delta SS_{\;},$$

where *C_ox_* is the oxide layer capacitance and Δ*SS* is the subthreshold swing variation.

The subthreshold swing (*SS*) is the change in gate voltage corresponding to each order of magnitude increase in subthreshold drain leakage current in the single logarithmic coordinate of the transfer curve. The calculation of *SS* is expressed by the following formula:

(4) $$SS=\frac{dV_{g}}{d(\log_{10}I_{d})}(V/\mathrm{dec})$$

where *V_g_* and *I_d_* are from the transfer curve of the devices.

**Figure 3:**
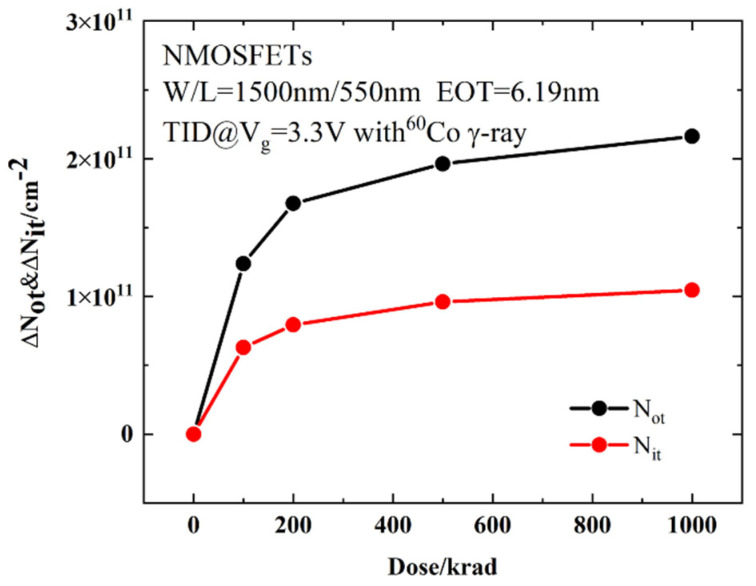
Δ*N_ot_* and Δ*N_it_* at different doses during radiation.

After calculation, the oxide layer charges and interface traps can be obtained, as shown in Figure 3.

It can be seen from Figure 3 that the oxide layer charges and interface traps increase rapidly before 200 krad (Si), and increase slowly after 200 krad (Si). The effect of oxide charges is greater than that of interface traps. For NMOSFETs, interface traps appear negatively charged and have a compensation effect on the positive charges generated by radiation. Therefore, even if the threshold voltage shifts caused by *N_ot_* and *N_it_* are large, the net shift of the NMOSFETs may be relatively small. As shown in Figure 3, the TID effect results in a relatively small negative shift of *V_th_* after radiation by the compensation effect of Δ*V_ot_* and Δ*V_it_*.

#### 3.2.2. Combined Effect of Radiation and Electrical Stress on Long-Term Reliability

**Figure 4:**
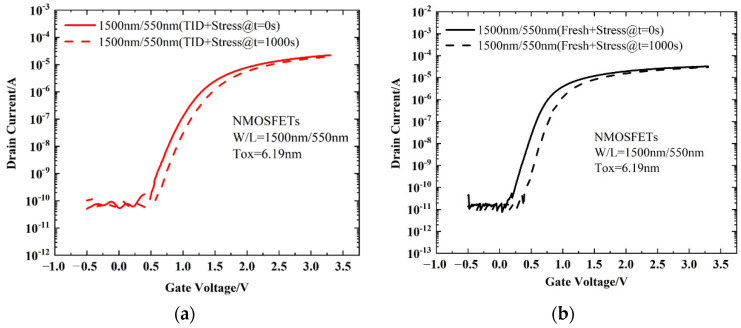
Drain current variations in *I_d_*–*V_g_* curves after electrical stress of (**a**) TID and (**b**) fresh devices. The *W*/*L* of the devices is 1.5 µm/0.55 µm.

**Figure 5:**
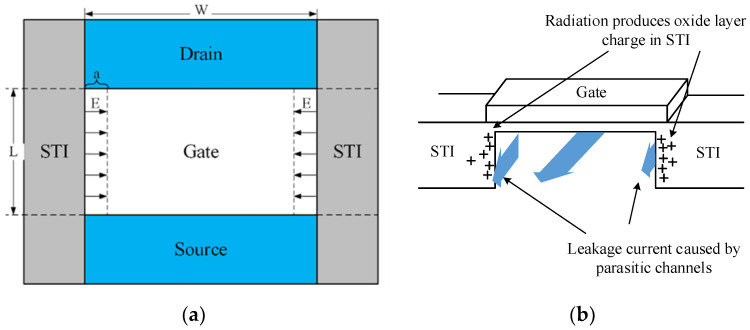
(**a**) Schematic diagram of STI electric field. (**b**) Schematic diagram of the parasitic channels generated by radiation in STI.

Figure 4 shows the variations in *I_d_*–*V_g_* curves after electrical stress of TID and fresh devices. As can be seen from Figure 4 the devices all shift positively after electrical stress. The shift of the *I_d_*–*V_g_* curve after TID + stress is less than that of the fresh + stress device, but the leakage current of the subthreshold is greater than that of fresh devices. The problem of increased leakage current is mainly caused by the oxide layer charge in STI. The effect of the positive charges in the STI cannot be ignored. Figure 5a shows the electric field at the STI. During the radiation process, the device not only generates positive charges in the gate oxide layer but also at the STI, generating an electric field at the STI, as shown in Figure 5a. Therefore, electrons can be attracted to the sides of the channel/STI and form two parasitic channels. The drain leakage current can be formed by electrons through the parasitic channels, as shown in Figure 5b. This makes the drain leakage current of the irradiated device increase greater than that of the non-irradiated device. The effect of STI is detailed in Section 3.3.

**Figure 6:**
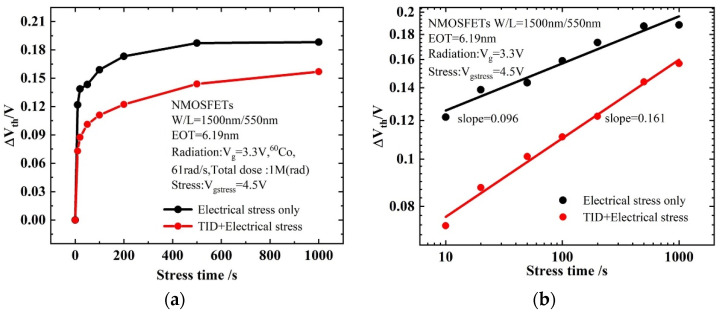
Degradation comparison of threshold voltage under electrical stress only and TID electrical stress: (**a**) linear coordinates; (**b**) double logarithm coordinates.

Under stress conditions, the threshold voltage is one of the most important degradation parameters. The threshold voltages are obtained from the transfer curves. It can be seen from Figure 6a that the threshold voltage degradation of the device which is applied a gate voltage stress after irradiation is smaller compared to that with a gate voltage applied directly. As can be seen in Figure 6b, in the double logarithmic coordinates, the threshold voltage shift shows a linear relationship to the stress time. In other words, the shift of the threshold voltage caused by the gate voltage stress conforms to the law of power function, similar to the effect of bias temperature instability, and its degradation expression is:

(5) $${\mathrm{lg}}_{\;}\left(\Delta V_{th}\right)=b+\alpha {\mathrm{lg}}_{\;}t,$$

**Figure 7:**
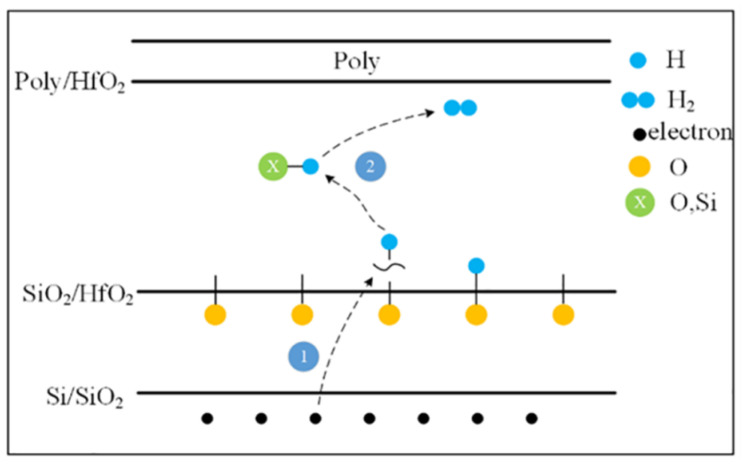
Mechanism of interface trap generation in a high-K dielectric.

Thus,

(6) $$\Delta V_{th}=10^{b}t^{\alpha },$$

where *α* is the slope of the linear fit in double logarithmic coordinates, and *b* is the curve intercept. In a non-irradiated device, the threshold voltage shifts by 0.2 V after 1000 s of stress; in an irradiated device, the threshold voltage shifts by 0.15 V after 1000 s of stress, which means that the radiation effect plays a role in weakening the degradation of the device during the biasing process. H will participate in both radiation and stress processes [20]. Unlike the reaction of H in the SiO_2_ layer of traditional MOSFETs [21], in high-K gate dielectrics, in addition to the Si/SiO_2_ interface, interface traps also occur at the SiO_2_/HfO_2_ interface. Figure 7 shows the mechanism of interface trap generation in a high-K dielectric.

When a positive gate bias stress is applied, the electrons in the channel inversion layer tunnel into the gate oxide layer, causing some Si-H or O-H bonds to break at the Si/SiO_2_ and SiO_2_/HfO_2_ interfaces, forming hydrogen atoms. Then, H atoms can react with X-H to form H_2_, thereby forming Si^−^ or O^−^ interface traps which appear negatively charged in NMOSFETs [22]. The model is as follows:

O-H + e^−^ = O^−^ + H; Si-H + e^−^ = Si^−^ + H, (7)

X-H + H = X^−^ + H_2_ (8)

where X-H is Si-H or O-H.

Furthermore, during the fabrication process, a large number of oxygen vacancy defects in HfO_2_ will be generated, and they act as electron trapping centers [23] so that electrons can be trapped in the oxide layer to form negative oxide layer charges [24].

As analyzed above, the interface traps and negative oxide charges can cause the threshold voltage to shift positively [25], and they have a strengthening effect on the devices. The final positive shift of *V_th_* is relatively large during the gate electrical stress.

**Figure 8:**
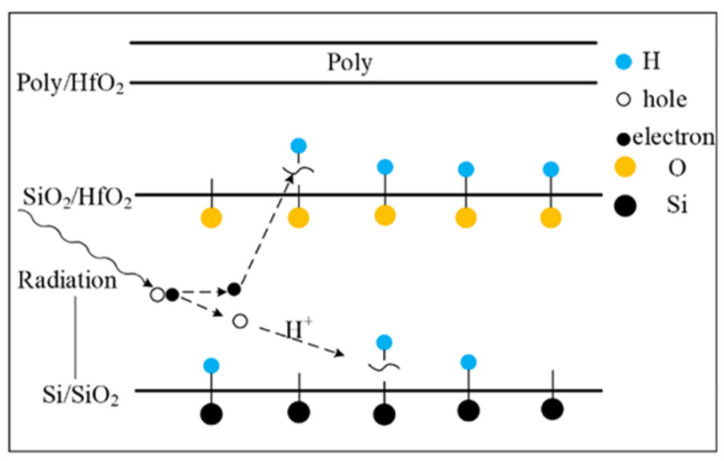
Radiation produces defects in the oxide layer.

It can be seen from Figure 8 that during the radiation process, the oxide layer will generate electron–hole pairs. Under the gate voltage, the electrons will move towards the HfO_2_ layer. Some of the electrons react with the Si-H bond at the SiO_2_/HfO_2_ interface and the HfO_2_ layer, consuming part of the H atoms. As a result, the H that can be reacted during the gate stress is reduced, and the degradation of the BTI-like effect is weakened. In addition, during the electrical stress experiment, a 4.5 V positive bias is applied to the gate, which will cause the electrons in the inversion layer to tunnel into the oxide layer. Since the radiation effect generates a large number of positive charges in the oxide layer, the tunneling electrons will neutralize part of the positive charges. This process consumes part of the electrons, thereby also weakening the BTI-like effect caused by gate stress. Based on the above two reasons, the degradation of devices with radiation combined with electrical stress is smaller than that of devices with only electrical stress, as shown in Figure 6.

Furthermore, the effects of the interface traps and oxide layer charges on the performance of the devices are significant [26]. With the varying of their densities, the threshold voltage shifts and the device reliability is affected. If the environmental condition such as radiation or electrical stress becomes more severe, the worse the device performance will be, and the more the reliability of the devices will degrade.

**Figure 9:**
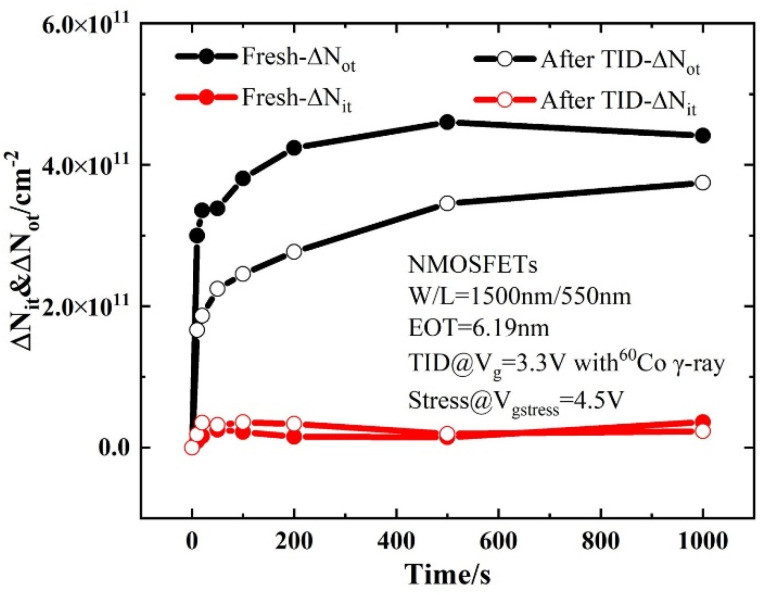
The relationship between Δ*N_it_* and Δ*N_ot_* and electrical stress time for the fresh devices and devices after TID.

Figure 9 shows the changes in interface traps and oxide charges (Δ*N_it_* and Δ*N_ot_*) with electrical stress time for fresh devices and devices after TID. Since the SiO_2_/HfO_2_ interface is inside the gate oxide layer, the defects generated by this interface can be regarded as oxide layer traps. In this way, the trap concentrations of Δ*N_it_* and Δ*N_ot_* can be calculated according to Equations (2) and (3), as shown in Figure 9. It can be seen that Δ*N_ot_* increases after stress for both devices, as the electrons are trapped in the oxide layer, forming oxidation layer charges. The Δ*N_it_* values of both devices are small, showing that the interface traps generated by the Si/SiO_2_ interface are small. The Δ*N_ot_* of the device after TID + stress is smaller than that of the fresh one, as part of H has been consumed during the radiation process, and the tunneling electrons will neutralize part of the positive charges generated by the radiation effect. Therefore, the final Δ*N_ot_* of the device after TID + stress is smaller.

### 3.3. Effect of Structure on Combined Effect of Radiation and Electrical Stress

#### 3.3.1. Effect of Gate Width and STI Parasitic Gate

**Figure 10:**
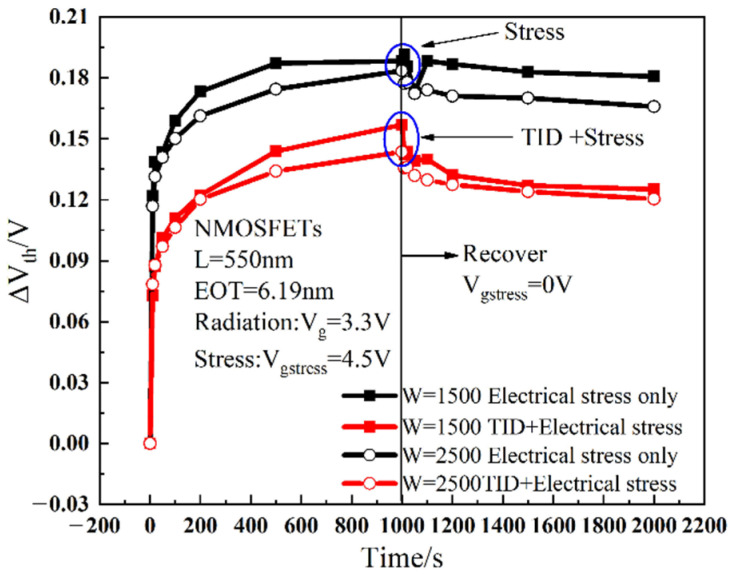
The relationship between threshold voltage shift with different gate widths as a function of time.

Figure 10 shows the relationship between the threshold voltage shift of different gate widths before and after radiation with time under electrical stress, where the stress time is 1000 s and the recovery time is 1000 s. It can be seen from Figure 10 that with the stress time accumulation, the NMOS devices show a certain degree of degradation, and the degradation trend gradually becomes saturated. The device will recover to a certain extent after 1000 s of recovery. This is because the gate stress is stopped and electrons stop tunneling, so the fracture process of the X-H bond stops, and the H ions diffuse back to the interface to passivate the X-H bond. As for the discussion before, the threshold voltage shift of the irradiated device is smaller than that of the non-irradiated device. Before and after radiation, the threshold voltage shifts are both larger during the stress process as the gate width becomes smaller, which is mainly related to the traps generated in STI by irradiation. In MOS devices, there are two kinds of oxide: gate oxide layer and shallow trench isolation (STI). With the thinning of the gate oxide, its influence on the device is reduced, and the influence of STI is highlighted [27]. In addition to the mechanical stress at the edge of the STI, there is also the electric field effect of the STI pointing to the channel due to the large number of positive charges generated in the STI region during radiation [28]. Therefore, the parasitic channel current of electrons is generated at both sides of STI and the parasitic gate effect is formed, making the threshold voltage shifts larger for devices with a narrower gate width.

**Figure 11:**
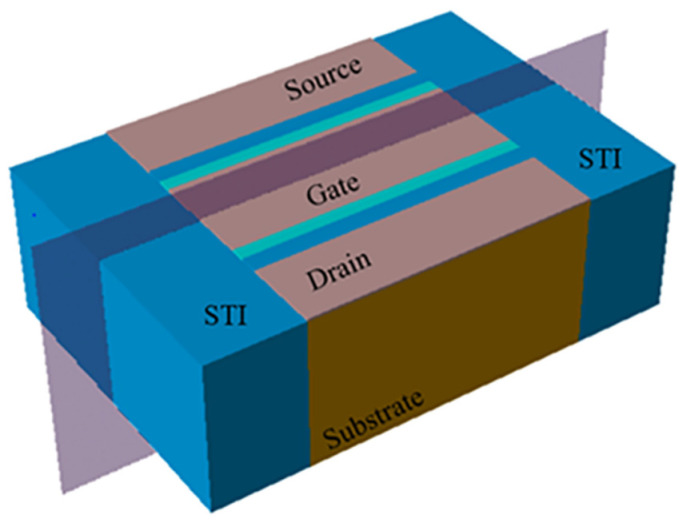
MOSFET simulation structure.

**Figure 12:**
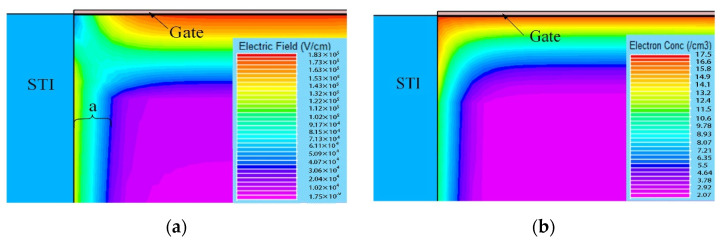
Device simulation results (STI in the gate width direction): (**a**) electric field and (**b**) electron density of the STI side.

In order to analyze the parasitic gate effect by STI, we simulated the physical characteristics of the STI side by SIVALCO. Figure 11 shows the three-dimensional simulation structure of the NMOS device. Figure 12a is a cross-sectional electric field diagram of the STI and the channel contact region along the gate width direction. As can be seen from Figure 12a, an electric field is generated at the interface between the STI and substrate silicon, pointing to the channel region from the STI region. Figure 12b shows the concentration of electrons generated at the substrate/STI interface due to the STI electric field, thereby forming a leakage channel. Then, the STI with positive charges by the TID effect can be seen as a parasitic gate. It can be seen from Figure 12a that as the gate width becomes smaller, the proportion 2a/W representing the influence of STI electric field regions increases. Therefore, under the same stress, the threshold voltage and other parameters of narrower devices degrade more.

#### 3.3.2. Effect of Gate Length and Gate Edge Effect

**Figure 13:**
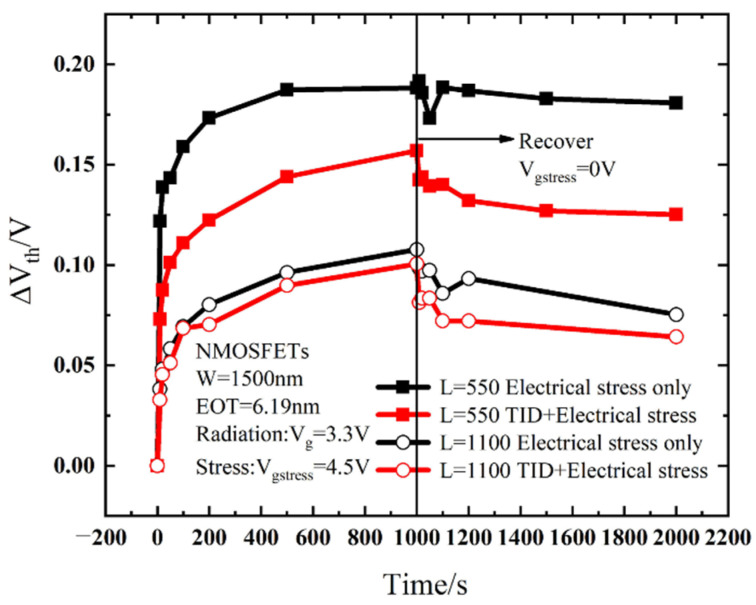
The relationship of threshold voltage shifts of devices with different gate lengths as a function of time.

**Figure 14:**
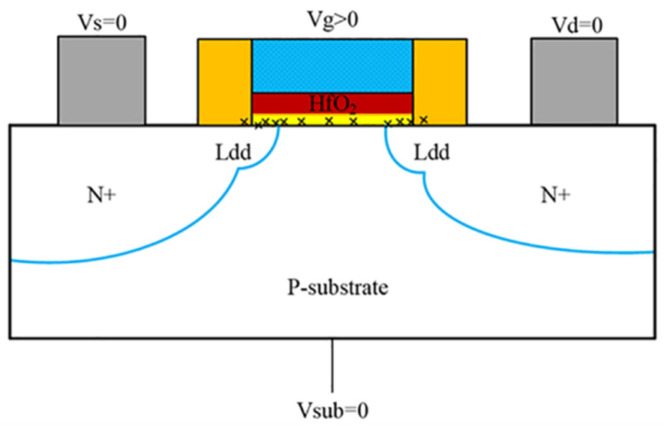
Schematic diagram of damage to the device non-uniformly distributed along the channel.

Figure 13 shows the relationship between the threshold voltage shift of devices with different gate lengths before and after radiation as a function of time. It can be clearly seen that as the gate length decreases, the degradation of device parameters increases significantly. Figure 14 is a schematic diagram of damage to the device non-uniformly distributed along the channel. Because the gate edge is close to the source and drain doping and the electron concentration is high, it will cause excessive diffusion of the H atoms in the Si_3_N_4_ layer [29,30]. The manufacturing process will cause more potential damage at the gate edge [31], and the trap density in the gate edge region is higher than that in the center of the channel. As the channel length decreases, the ratio of the gate edge region to the total gate length increases, and the influence of the gate edge effect increases. Therefore, the dependence of the threshold voltage degradation of the device on the gate length is the same as that shown in Figure 13.

## 4. Conclusions

We studied the reliability of the combined effect of TID and electrical stress on NMOSFETs. First, the combined effect of radiation and electrical stress makes the degradation of threshold voltage and other characteristic parameters smaller, and the radiation effect weakens the ultimate degradation of the device. The electrons introduced by the radiation effect can destroy part of the H bonds, and the H bonds are an important reason for the degradation of the device during the stress process. Therefore, the degradation of the BTI-like effect is weakened. During the application of positive gate stress, the electrons tunnel into the oxide layer and neutralize part of the positive charges generated during the radiation process, which also reduces the degradation of the BTI-like effect. Secondly, the radiation effect will generate parasitic gates in the STI region, and the influence of the parasitic gates will increase as the gate width decreases. The gate edge region has more potential damage, resulting in a higher defect density than the middle region of the channel. The influence of the gate edge effect will increase as the gate length decreases. Therefore, the degradation of the device will be larger with a narrower and shorter gate.
